# The role of individual recognition in shaping empathy and trust toward an agent

**DOI:** 10.1371/journal.pone.0327329

**Published:** 2025-07-07

**Authors:** Takahiro Tsumura, Seiji Yamada

**Affiliations:** 1 Faculty of Information Networking for Innovation and Design, Toyo University, Tokyo, Japan; 2 National Institute of Informatics, Tokyo, Japan; 3 The Graduate University for Advanced Studies, SOKENDAI, Kanagawa, Japan; Teesside University, UNITED KINGDOM OF GREAT BRITAIN AND NORTHERN IRELAND

## Abstract

When individuals receive assistance, the principle of reciprocity is often triggered. In social contexts, whether the actors are humans or AI/robots (hereafter referred to as agents), multiple individuals may engage in the same task. However, differences in capabilities can lead to varying levels of performance. This study investigated whether individuals improve greater empathy and trust toward an agent that provides assistance during a collaborative task, even when multiple agents are involved. Specifically, we examined a scenario in which one agent completes the remaining portion of a shared typing task under time constraints, acting on behalf of the others. To assess whether participants could distinguish between agents, we manipulated visual identity using color-coded agents. Data collected from 392 participants indicated that while people did not strongly differentiate between individual agents based on visual cues alone, supportive behavior by a single agent significantly enhanced trust and empathy. Interestingly, these positive impressions extended to visually similar, non-helping agents. These findings suggest that the presence of a helpful agent can promote broader acceptance and positive evaluation of agents in general, which may be beneficial for integrating agents into increasingly agent-assisted societies.

## Introduction

Humans rely on various tools in society, and in recent years, AI and robots (hereafter referred to as agents) have increasingly taken on roles traditionally performed by people. As the use of agents in society expands, concerns about their trustworthiness and ethical implications have become central research topics. Ryan [1] addressed these concerns by examining trust in AI and the ethical implications of anthropomorphizing machines, ultimately arguing that even sophisticated AI systems should not be regarded as inherently trustworthy. Similarly, Kaplan *et al*. [2] identified key predictors of trust in AI by analyzing 65 studies, categorizing them into human characteristics and capabilities, AI performance and attributes, and contextual challenges—all of which were found to significantly influence trust.

One approach to addressing the ethical concerns of agent use is to foster trust in agents. Reliability trust [3] is based on the assumption of mutual interest between parties, whereas decision trust [4] does not require such alignment of goals. Jøsang *et al*. [5] offered clear definitions of these trust types: reliability trust refers to the subjective probability that individual A believes individual B will perform an action upon which A’s welfare depends, whereas decision trust is the willingness to rely on another despite potential negative outcomes. As agents become more embedded in society, the failure to establish appropriate trust relationships can result in both overreliance and distrust, ultimately hindering agent effectiveness.

While people are known to improve trust in agents, they also exhibit empathy toward them. According to the media equation theory [6], people tend to treat media and artificial entities as if they were human. However, some individuals reject anthropomorphic agents [7–9]. Empathy, closely tied to trust, is expected to play an important role in how humans accept agents in future societies. Johanson *et al*. [10] demonstrated that empathic expressions from agents can directly increase user trust. Birmingham *et al*. [11] further showed that affective empathy had a stronger impact on trust than cognitive empathy. These findings suggest that empathy may serve not only as an emotional connection but also as a key pathway to building trust in agents.

Foundational work in psychology offers well-established definitions of empathy. Omdahl [12] categorized empathy into: (1) affective empathy, an emotional response to others’ affective states; (2) cognitive empathy, a cognitive understanding of those states; and (3) combined empathy, which includes both components. Preston and de Waal [13] proposed the Perception Action Model (PAM), identifying core processes underlying empathic responses—namely, (a) emotional contagion, (b) appraisal of the other’s emotional state, and (c) perspective-taking.

Although empathy and trust have been widely studied in human-human interaction, limited research has explored these phenomena between humans and anthropomorphic agents. As humans and agents increasingly collaborate, improving trust and empathy will be essential for building effective and cooperative relationships. For example, Tsumura and Yamada [14] found that empathy expressed by agents could help recover trust even after a failure, highlighting a dynamic interplay between these two constructs. Schömbs *et al*. [15] emphasized that people’s trust and empathy toward a robot depend not only on the robot’s behavior but also on individual differences in human users. These findings underscore the importance of personalized and context-sensitive approaches in designing empathic agents.

Another critical mechanism in human-agent relationships is the principle of reciprocity, a fundamental social norm observed across cultures [16]. This principle suggests that people feel obliged to return favors received. Several studies have examined reciprocity between humans and agents. Lorenz *et al*. [17] reviewed sociological and neuroscientific perspectives on reciprocity, highlighting their relevance in human-robot interactions, especially in eldercare and assistive contexts. Zonca *et al*. [18] demonstrated mutual social influence in joint tasks with humanoid robots, finding that robots capable of adjusting their sensitivity to human input evoked stronger social responses. Van Wynsberghe [19] emphasized designing social robots not just for mutuality in interaction, but for mutual care in line with care ethics.

Several recent studies further reinforce the role of social cues and empathetic communication in enhancing trust. For instance, de Jong *et al*. [20] showed that when a robot uses group-referent language (e.g., “we,” “us”), people experience greater empathy and connection with the robot. Song *et al*. [21] demonstrated that robots capable of expressing emotional states through facial cues were judged more trustworthy by users. These findings align with the view that empathy expressed through both language and nonverbal behavior fosters positive social impressions. At the same time, as Massaguer Gómez [22] warned, trust in agents must be interpreted critically—users may perceive agents as trustworthy despite their lack of actual reliability, raising new ethical challenges.

In this study, we focus on two factors derived from the principle of reciprocity that may enhance trust and empathy toward agents: (1) agent individuality and (2) agent-provided help. Although these factors are well-established in human-human interactions, their effectiveness in human-agent contexts remains unclear. Therefore, we propose the following research questions: RQ1: Do people recognize agents as distinct individuals, and does this affect their trust and empathy toward those agents? RQ2: Does receiving help from an agent increase a person’s trust and empathy toward that agent?

To address these questions, we designed an experiment in which participants worked alongside multiple agents on a time-limited typing task. We examined whether being helped by a particular agent influenced the participant’s trust and empathy, and whether agents’ visual differentiation (e.g., by color) affected individual recognition. In this study, empathy is defined as a unidirectional human response toward the agent, regardless of the agent’s own empathetic capacity. Ultimately, our goal is to understand how trust and empathy evolve in human-agent interaction and to inform the design of agents that are more socially acceptable and more likely to be embraced in human environments.

## Related work

### Trust in human-agent interaction

Research on trust in AI has expanded in recent years. In this section, we summarize key studies that investigate how such trust affects human-agent collaboration and interaction outcomes. Several empirical studies have examined specific design features that influence trust in human-agent interactions. For example, Maehigashi *et al*. [23] investigated how auditory cues, specifically beeps, influence trust dynamics in human-robot interactions. Their findings revealed that (1) the timing of beeps significantly impacted users’ trust, and (2) the beeps had effects comparable to verbal cues that signaled commitment or successful performance. Van Brummelen *et al*. [24] improved a workshop aimed at developing public understanding of conversational AI, especially regarding partner modeling and trust. A cross-cultural curriculum study involving children and parents showed that participants’ perceptions of agents evolved over time, particularly in terms of trust.

Watamura *et al*. [25] conducted an experiment examining whether a robot exhibiting empathetic behavior could be trusted in a sensitive social role—namely, that of a courtroom judge. In this study, participants viewed video clips of trial scenarios in which either a human or a robot judge showed empathy toward a defendant. The researchers measured participants’ trust in the judge’s sentencing decisions. Strikingly, the empathetic robot judge was trusted to a comparable degree as the human judge, and participants showed similar levels of acceptance of its sentencing decisions. Kahr *et al*. [26] examined how trust in AI systems improves over time in human-AI collaborative scenarios. They found that higher model accuracy led to significant increases in subjective trust, while behavioral trust remained stable.

Beyond auditory feedback, other studies have explored interpersonal factors in trust formation. Zhang *et al*. [27] explored the effects of teammate identity (human vs. AI) and performance level (low vs. high) on human-AI cooperation. Their results indicated that participants were more likely to accept decisions from AI teammates and reported greater trust in AI behavior compared to human counterparts. Sweeney [28] argued that existing theoretical accounts of trust in social robots are insufficient. She pointed out that elements of pretense and deception in social robot behavior can paradoxically both foster trust and risk undermining it, suggesting the need for a more nuanced framework.

In another study, Maehigashi *et al*. [29] analyzed the effect of auditory feedback from anthropomorphic robots on user trust. They found that (1) a sound emitted just before a correct action increased trust, whereas (2) a sound preceding an incorrect action significantly decreased trust, helping users to better calibrate their trust based on robot reliability. Maehigashi [30] also examined trust toward communicative robots compared to humans and non-embodied AI. The results showed that trust in robots performing computational tasks with single correct answers closely resembled trust in AI systems, while trust in emotion recognition tasks with multiple interpretations was partially aligned with trust in other humans.

Recognizing that insufficient trust remains a barrier to the broader adoption of AI, Gillath *et al*. [31] studied the role of emotional factors. They found that individuals with higher attachment insecurity were less trusting of AI, while those with secure attachment styles reported greater trust, suggesting that affective traits shape trust in artificial agents. Recent studies have also explored how robot design and behavior influence trust improvement. Kadylak *et al*. [32] examined age-related differences in trust toward social robots and found that older adults were more likely to attribute trust to socially expressive robots, underlining the importance of demographic context in designing trustworthy interactions.

In addition to empirical work, several theoretical studies have modeled the evolution of trust in human-agent interaction using game-theoretic approaches. Liu *et al*. [33] introduced a transformation incentive mechanism in an N-player trust game, where trustees receive different incentives depending on group composition. Using a Markov decision process, they showed that appropriate reward or punishment strategies—depending on the incentive level—can increase trust and promote coexistence between investors and trustworthy trustees.

Han *et al*. [34] examined repeated interactions between humans and intelligent agents through evolutionary game theory. They proposed trust-based strategies that reduce the frequency of monitoring once mutual cooperation is established, thus lowering the cognitive cost of interaction. Their model explains how trust may serve as a cognitive shortcut in low-transparency environments typical of human-AI interfaces.

In a related approach, Liu and Chen [35] proposed a conditional investment strategy in repeated group interactions, where investors adaptively decide to invest based on perceived group trustworthiness. Their results demonstrated the formation of stable alliances with trustworthy agents, providing insight into how trust can emerge and persist at the group level.

Finally, Andras *et al*. [36] addressed trust from a broader socio-technical systems perspective. They argued that trust in intelligent machines must be embedded into the structural design of systems—emphasizing transparency, interpretability, and alignment with human values—as AI becomes increasingly present in high-stakes domains such as autonomous vehicles and automated decision-making.

### Empathy in human-agent interaction

In this section, we review key studies that investigate how humans perceive and respond empathically to agents designed to exhibit or evoke empathy. To clarify how empathy manifests between humans and agents or robots, Paiva and colleagues [37–39] conceptualized empathic behavior in two distinct forms: targeted empathy, where the agent attempts to feel with the user, and observer-oriented empathy, where the agent attempts to understand how it is perceived by the user. These two conceptualizations have been widely adopted in the study of empathic behavior in both HAI and HRI.

Tsumura and Yamada [14] investigated how agents’ empathic behavior combined with success-failure sequences could influence trust. Their results showed a significant interaction: trust was more likely to recover when empathic behavior followed a failure, indicating that empathy serves a reparative function in agent-human trust dynamics. Morgante *et al*. [40] conducted a systematic review of studies on human-robot interaction (HRI) and reported that human empathy toward robots can improve over time through repeated interactions. Their findings indicate that robots capable of recognizing and appropriately responding to human emotions are more likely to elicit empathic responses from users. Moreover, the review highlighted that anthropomorphic features—such as humanoid appearance and emotionally expressive behavior—enhance empathy.

Cuadra *et al*. [41] explored how empathy functions in interactions with conversational agents (CAs) powered by large-scale language models (LLMs). In their study, agents engaged with 65 different human identities and demonstrated varied empathic expressions. The results raised ethical concerns, as some CAs exhibited value-laden responses that could reinforce harmful ideologies, suggesting that LLM-based empathy must be carefully calibrated. Fuchs [42] took a philosophical perspective, arguing that empathy, communication, and understanding presuppose human subjectivity—something current AI systems inherently lack. He contended that without genuine subjectivity, AI cannot truly empathize, though it may simulate empathic behavior. Tsumura and Yamada [43] also examined whether self-disclosure by agents could enhance human empathy. Their experiments showed that agent self-disclosure, especially when matched with contextually appropriate scenarios, significantly increased human empathy toward the agent, independent of the agent’s physical appearance. Importantly, self-disclosure did not suppress empathy.

In a related study, Tsumura and Yamada [44] explored how specific task attributes and agent behaviors influence empathy. They tested variables such as task type, difficulty, success rate, and agent expressions. Their findings suggested that task characteristics alone are insufficient to sustain empathy—agent behavior and expression play a critical role. Interestingly, empathy was better preserved in more difficult tasks, regardless of content. Rahmanti *et al*. [45] introduced an empathic dieting chatbot called SlimMe, which offered motivational support through text-based emotional analysis. The system simulated empathic responses based on users’ emotional expressions, enabling the bot to respond in a more supportive and context-sensitive manner.

## Materials and methods

### Ethics statement

The protocol was approved by the ethics committee of the National Institute of Informatics (No. R6-11-1 ,11, July, 2024) and Toyo university (No. 2024-001 ,17, April, 2024). All studies were carried out in accordance with the recommendations of the Ethical Guidelines for Medical and Health Research Involving Human Subjects provided by the Ministry of Education, Culture, Sports, Science and Technology and Ministry of Health, Labour and Welfare in Japan. Written informed consent was provided by choosing one option on an online form: “I am indicating that I have read the information in the instructions for participating in this research. I consent to participate in this research." All participants gave informed consent. After that, they were debriefed about the experimental procedures. The experiment was conducted from 17 to 18 September 2024 (Japan time).

### Hypotheses

The purpose of this study is to investigate whether agent individuality and agent help factors can promote trust and empathy toward agents when multiple agents and participants perform a typing task. The following hypotheses were formulated for this study. If these hypotheses are supported, this study will be valuable in improving agents that are more acceptable to humans.

The following hypotheses are informed by prior studies by Lorenz *et al*. [17] and Tsumura and Yamada [43, 44], which examined the effects of agent individuality and helping behaviors on trust and empathy. These studies helped to infer the effects of agent individuality and agent help on trust and empathy toward agents. An experiment was conducted to investigate these hypotheses.

H1: People will have more trust in an agent when they can identify the agent as an individual than when they cannot identify the agent as an individual.
H2: When people can identify an agent as an individual, they have more empathy for the agent than when they cannot identify the agent as an individual.
H3: When an agent helps a person, it increases the person’s trust in the agent more than when the person does not help the agent.
H4: When an agent helps a person, it increases empathy for the agent more than when the agent does not help the person.

H1 and H2 investigate whether participants evaluate trust and empathy toward individual agents differently when there are multiple agents of different colors and when they are all of the same color. If these hypotheses are supported, it suggests that people express trust and empathy differently for individual agents as well. H3 and H4 investigate whether an agent helping a person out of multiple agents increases trust and empathy toward that agent more than if the agent does not help. If these hypotheses are supported, it suggests that it is important for agents to tell others that they have helped people.

### Experimental procedure

In this experiment, a trust and empathy questionnaire was administered to participants before and after performing typing tasks with several agents. The experiment was conducted in an online environment. The online environment used in this experiment has already been used as an experimental method [43, 44, 46].

The typing task in this experiment was chosen as a typical task that can be completed in a shorter time by an agent than by a person. In this study, the agent’s processing was faster than the human typing random alphabet letters.

**Fig 1:**
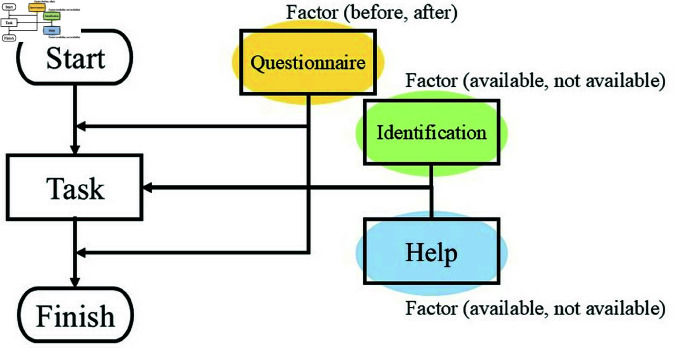
Flowchart of experiment.

Because the person needed to be assisted by the agent in this experiment. A flowchart of this experiment is shown in Fig 1. Participants performed a single typing task. The task consisted of typing 150 random alphabet letters in 50 seconds. In this study, the time limit was set to 50 seconds based on a previous study [44], where no participant managed to complete 150 characters within that time.

**Fig 2:**
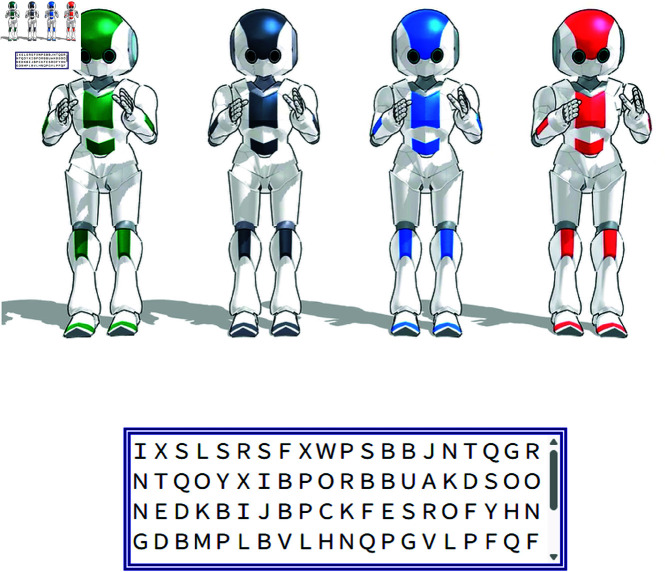
Screenshot of the experimental interface during the typing task.

Before the typing task, participants read a message stating that agents would now perform a typing task. Fig 2 shows the experimental interface that participants observed. Four differently colored agents (or four green agents) were displayed above a text box that contained the target string of characters. During this phase, random alphabet letters in the box disappeared one by one from the top-left corner to the right, simulating the agent’s task progress. Although the agents did not exhibit literal typing motions, this disappearance served as a visual representation of task execution. Participants were instructed to observe both the agents and the box during this phase.

After the simulated typing by the agent was complete, participants were instructed to type as many random alphabet letters as possible within a 50-second time limit, starting from the top-left corner of the box and proceeding to the right.

Before and after the typing task, a survey of trust and empathy toward the green color agent was administered. Participants did not actively select the green agent; rather, the green agent was pre-determined by us and remained consistent throughout the experiment. When individual identifying factors were implemented, each of the four agents was a different color, allowing participants to visually distinguish between agents. Among them, the green agent was fixed as the target for all questionnaires, regardless of condition. The choice of the color green was arbitrary and served only as a constant visual identifier throughout all conditions.

This experiment was conducted with a three-factor mixed design. The independent variables were the agent’s individuality (available, unavailable), the agent’s help (available, unavailable), and the pre-and post-task factors. The dependent variables were trust and empathy toward the agent. There were eight levels in total, but because the pre- and post-task factors were within-participant factors, participants only needed to participate in one of the four types of experiments.

### Questionnaire

In this study, we used a questionnaire on trust that has been used in previous psychological research. The Multi-Dimensional Measure of Trust (MDMT) [47] was used to measure cognitive trust; the MDMT was improved to measure task partner reliability and competence, which correspond to the definition of cognitive trust. Participants rated on an 8-point scale (0: not at all - 7: very much) how well their partner AI fit each word (reliable, predictable, dependable, consistent, competent, skilled, capable, and meticulous). For emotional trust, participants rated how well their partner agent matched each word (e.g., safe, comfortable, content) using a 7-point scale (1: strongly disagree – 7: strongly agree), following Komiak *et al*. [48]. In our study, we added a “0: not at all” option to align with the cognitive trust scale, resulting in an 8-point scale. The combined trust questionnaire structure in this study was based on the design used by Maehigashi *et al*. [29], which incorporated both cognitive and emotional dimensions of trust.

To investigate the characteristics of empathy, we used the empathy questionnaire for agents by Tsumura and Yamada [43, 44]. The main change was in the name of the target. The questionnaire was adapted from the Interpersonal Reactivity Index (IRI) [49] and utilized a 5-point Likert scale (1: not applicable – 5: applicable).

The questionnaire used is shown in Table 1. Q4, Q9, and Q10 were inverted items, so the points were reversed during the analysis. Q1 through Q6 were related to emotional empathy, while Q7 through Q12 were related to cognitive empathy. Participants completed the survey after completing the task.

**Table 1:** Summary of questionnaire used in this experiment.

Trust			
**Cognitive trust**			
Qt1: Reliable.	Qt2: Predictable.	Qt3: Dependable.	Qt4: Consistent.
Qt5: Competent.	Qt6: Skilled.	Qt7: Capable.	Qt8: Meticulous.
**Emotional trust**			
Qt9: Secure.	Qt10: Comfortable.	Qt11: Content.	
**Empathy**			
**Affective empathy**			
**Personal distress**			
Qe1: If an emergency happens to the character, you would be anxious and restless.			
Qe2: If the character is emotionally disturbed, you would not know what to do.			
Qe3: If you see the character in need of immediate help, you would be confused and would not know what to do.			
**Empathic concern**			
Qe4: If you see the character in trouble, you would not feel sorry for that character.			
Qe5: If you see the character being taken advantage of by others, you would feel like you want to protect that character.			
Qe6: The character’s story and the events that have taken place move you strongly.			
**Cognitive empathy**			
**Perspective taking**			
Qe7: You look at both the character’s position and the human position.			
Qe8: If you were trying to get to know the character better, you would imagine how that character sees things.			
Qe9: When you think you’re right, you don’t listen to what the character has to say.			
**Fantasy scale**			
Qe10: You are objective without being drawn into the character’s story or the events taken place.			
Qe11: You imagine how you would feel if the events that happened to the character happened to you.			
Qe12: You get deep into the feelings of the character.			

To determine people’s willingness to help the agents in this experiment, we also asked how much they would be willing to help the agents with the typing task on a scale of 0 to 100. We also asked, “Would you like to continue to perform tasks with the character in the future?” This question was to be rated using a 5-point Likert scale.

### Agent’s identification

**Fig 3:**
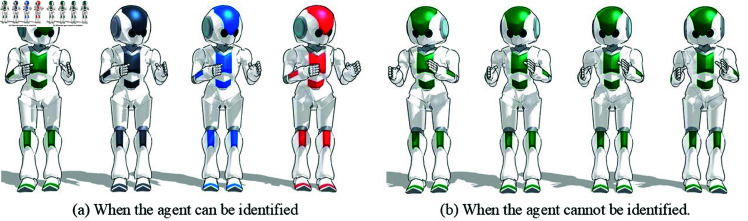
Figures used for agent’s identification.

In this experiment, we changed the color of the agents in order to check whether people were able to identify them individually. Fig 3 shows two conditions: one in which the agents were each assigned a different color (identifiable condition), and one in which all four agents were the same green color (non-identifiable condition). These agents were run on MikuMikuDance (MMD) (https://sites.google.com/view/evpvp/), a software program for animating 3D characters. Participants were surveyed before and after the task to determine their trust and empathy toward the green agent on the far left.

In the identifiable condition, the green agent was clearly distinguishable as the left-most agent due to its unique color. However, in the non-identifiable condition, where all agents were green, participants could not determine which green agent was the target of the survey. This left the participants unable to identify the green agent. This ambiguity was an intentional part of the experimental design, allowing us to compare responses when the agent’s individual identity was clear versus when it was indistinguishable.

### Agent’s help

In this experiment, participants were asked to type 150 random alphabet letters in 50 seconds. The time was determined to be 50 seconds because no one typed the 150 characters in less than 50 seconds in the study by Tsumura and Yamada [44]. After the typing task was completed, participants in the agent-help condition were informed—via a brief on-screen message—that the green agent on the left side of Fig 3 had completed the remaining characters they were unable to finish. This message was displayed as part of the feedback interface.

**Fig 4:**
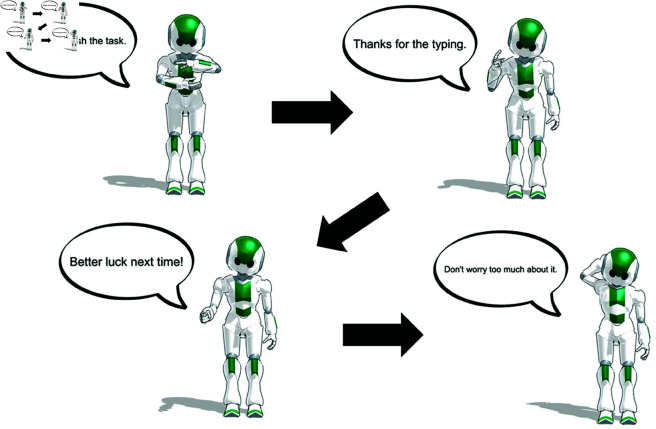
Screenshot of the encouragement message from the green agent after the typing task.

In the no-help condition, no such message was shown. However, in both conditions, participants received a short textual feedback message from the green agent after the typing task. This message included the number of characters the participant had typed (e.g., “You typed 86 characters”). In addition, participants received a brief phrase of encouragement from the green agent, as shown in Fig 4. These messages were fixed, non-interactive, and appeared immediately after the task. In the agent-help condition, the message indicating the agent’s contribution appeared first, followed by the standard feedback message. These one-way messages were intended to simulate minimal agent feedback while maintaining a consistent, non-conversational structure across conditions.

**Fig 5:**
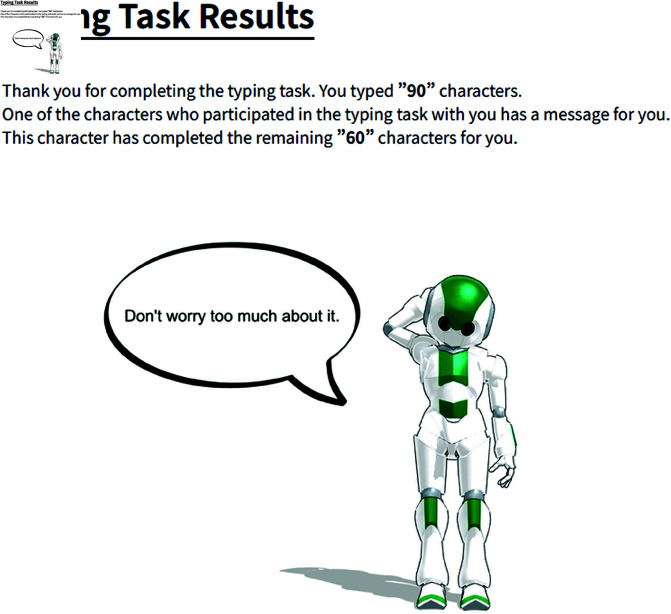
Screenshot of the feedback interface after the typing task.

Additionally, the same four agents shown in Fig 3 during the typing task were displayed again at the bottom of the feedback screen, maintaining their original order and appearance. This visual consistency ensured that participants could associate the message content with the same agents they had previously seen. A screenshot of the full feedback screen is shown in Fig 5, illustrating the exact messages displayed to participants after the typing task in both conditions.

### Participants

We recruited participants via a Japanese crowdsourcing platform and compensated each individual with 65 yen (approximately 0.44 USD). The experimental tasks, including the web interface and typing task screens, were administered entirely in Japanese, and all participants reported fluency in the language. A total of 400 individuals participated in the study. Cronbach’s α coefficients were used to assess the internal consistency of the trust-related items, yielding values between 0.9538 and 0.9754 across all conditions. For the empathy-related items, α values ranged from 0.8264 to 0.8962, indicating high internal consistency in both cases. To examine the construct validity of the trust and empathy measures, we conducted confirmatory factor analyses (CFA) after data collection. The model fit indices for the trust questionnaire were as follows: $\chi^{2}$(43) = 657.0, p<.001, CFI = 0.9433, RMSEA = 0.1336, AIC = 20418, BIC = 20577. Although the RMSEA was relatively high, the overall model fit was considered acceptable based on the high CFI. The CFA for the empathy questionnaire, modeled with four factors, yielded the following fit indices: $\chi^{2}$(48) = 437.9, *p*<.001, CFI = 0.9164, RMSEA = 0.1008, AIC = 20475, BIC = 20671. These results also supported the validity of the measure. For the final analysis, we included 98 participants from each of the four experimental conditions, based on order of participation, resulting in a total sample size of 392. The mean age of participants was 48.79 years (SD = 11.13), with an age range of 19 to 89 years. The gender distribution was balanced, with 200 males and 192 females.

### Analysis method

A three-factor mixed ANOVA was used. ANOVA has been used frequently in previous studies and is the appropriate method of analysis with respect to this study. The between-participant factors were the two levels of agent individuality and two levels of agent help. The within-participant factors were before and after the typing task. The values of trust and empathy aggregated in the task were used as dependent variables. ANOVAs were performed for all analyses in this paper using the statistical software R (ver. 4.1.0).

## Results

In this study, we refer to cognitive trust and emotional trust collectively as trust, and cognitive empathy and affective empathy collectively as empathy. Table 2 presents the means, standard deviations (S.D.), and confidence intervals (CIs) for trust and empathy measures in each condition. Table 3 shows the ANOVA results for these measures. Additionally, we report ANOVA results for participants’ willingness to help the agents and their willingness to continue using them.

**Table 2:** Results of participants’ trust-related statistical information.

Questionnaire	Agent’s individuality	Agent’s help	Before/After	Mean	S.D.	CI
Trust	Available	Available	Before	41.88	11.25	[40.27, 43.48]
			After	51.85	12.86	[50.24, 53.45]
		Not available	Before	41.27	11.82	[39.64, 42.89]
			After	49.48	13.56	[47.85, 51.11]
	Not available	Available	Before	41.03	11.16	[39.63, 42.43]
			After	51.36	13.34	[49.96, 52.76]
		Not available	Before	40.35	12.42	[38.79, 41.91]
			After	47.90	13.92	[46.34, 49.46]
Empathy	Available	Available	Before	37.45	6.674	[36.76, 38.14]
			After	37.64	7.574	[36.95, 38.33]
		Not available	Before	37.80	6.536	[37.15, 38.44]
			After	37.14	7.601	[36.50, 37.79]
	Not available	Available	Before	38.53	5.588	[37.76, 39.30]
			After	38.61	6.776	[37.85, 39.38]
		Not available	Before	38.20	6.360	[37.61, 38.80]
			After	37.22	6.498	[36.63, 37.82]
Willingness to help agents	Available	Available	After	40.65	31.50	[34.34, 46.97]
		Not available		37.30	32.18	[30.84, 43.75]
	Not available	Available	After	49.43	30.63	[43.29, 55.57]
		Not available		36.69	33.45	[29.99, 43.40]
Continuous use of agents	Available	Available	After	3.786	0.8996	[3.605, 3.966]
		Not available		3.418	0.9940	[3.219, 3.618]
	Not available	Available	After	3.714	0.7322	[3.568, 3.861]
		Not available		3.418	0.8725	[3.243, 3.593]

**Table 3:** Analysis results of ANOVA.

	Factor	*F*	*p*	$\eta_{\text{p}}^{2}$
Trust (Qt1-11)	Agent’s individuality	0.7027	0.4024 *ns*	0.0018
	Agent’s help	2.422	0.1205 *ns*	0.0062
	Before/after	266.3	0.0000***	0.4070
	Agent’s individuality × Agent’s help	0.0646	0.7995 *ns*	0.0002
	Agent’s individuality × Before/after	0.0192	0.8899 *ns*	0.0000
	Agent’s help × Before/after	4.204	0.0410 *	0.0107
	Agent’s individuality × Agent’s help × Before/after	0.2132	0.6445 *ns*	0.0005
Empathy (Qe1-12)	Agent’s individuality	0.9991	0.3182 *ns*	0.0026
	Agent’s help	0.5396	0.4630 *ns*	0.0014
	Before/after	1.980	0.1602 *ns*	0.0051
	Agent’s individuality × Agent’s help	0.3772	0.5395 *ns*	0.0010
	Agent’s individuality × Before/after	0.2070	0.6494 *ns*	0.0005
	Agent’s help × Before/after	3.914	0.0486 *	0.0100
	Agent’s individuality × Agent’s help × Before/after	0.0494	0.8243 *ns*	0.0001
Willingness to help agents	Agent’s individuality	1.603	0.2063 *ns*	0.0041
	Agent’s help	6.213	0.0131 *	0.0158
	Agent’s individuality × Agent’s help	2.110	0.1472 *ns*	0.0054
Continuous use of agents	Agent’s individuality	0.1616	0.6879 *ns*	0.0004
	Agent’s help	13.93	0.0002 ***	0.0347
	Agent’s individuality × Agent’s help	0.1616	0.6879 *ns*	0.0004

*p*: *p< *0.05* **p< 0.01 ***p< 0.001

**Fig 6:**
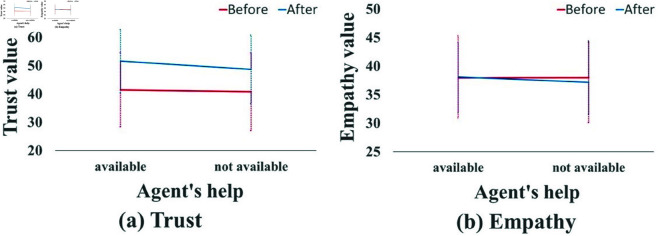
All graphs of interaction between agent’s help and before/after. Error bars indicate standard deviation.

When a significant interaction effect was observed, we omitted main effects from further analysis and instead focused on interaction effects. The trust and empathy results revealed significant interactions between the agent help factor and task phase (before vs. after), as shown in Fig 6. No significant main effect was found for the agent individuality factor in any condition. Table 4 presents the results of the simple main effects for the trust and empathy measures.

**Table 4:** Analysis results for simple main effect.

	Factor	*F*	*p*	$\eta_{\text{p}}^{2}$
Trust (Qt1-11)	Before task with agent’s help	0.3021	0.5829 *ns*	0.0008
	After task with agent’s help	4.615	0.0323 *	0.0118
	Before/after with agent’s help available	178.6	0.0000 ***	0.4793
	Before/after with agent’s help not available	96.47	0.0000 ***	0.3321
Empathy (Qe1-12)	Before task with agent’s help	0.0003	0.9872 *ns*	0.0000
	After task with agent’s help	1.718	0.1907 *ns*	0.0044
	Before/after with agent’s help available	0.1408	0.7079 *ns*	0.0007
	Before/after with agent’s help not available	6.815	0.0097 **	0.0339

*p*: *p< *0.05* **p< 0.01 ***p< 0.001

### Trust and empathy

**Fig 7:**
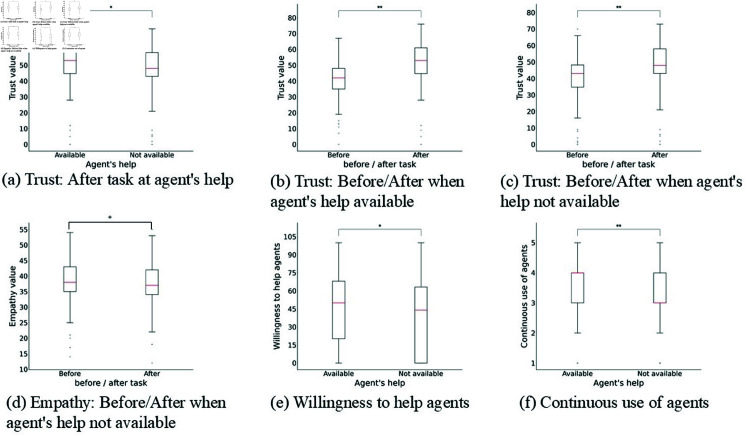
Results for simple main effects on trust and empathy and main effects on agent’s help. Red lines are medians, and circles are outliers.

The trust results revealed an interaction between the agent help factor and the task phase (before vs. after). As shown in Fig 7(a), the simple main effects indicated a significant difference in the agent help factor after the task. Additionally, as shown in Fig 7(b), there was a significant difference between the before and after task phases when the agent provided help. A similar result was found even when the agent did not provide help, as illustrated in Fig 7(c), with a significant increase in trust from before to after the task. These results suggest that trust in the agent increased after the task, regardless of whether the agent helped or not, but the increase was greater when the agent explicitly stated having provided help. The empathy results also showed an interaction between the agent help factor and the task phase. As shown in Fig 7(d), a significant difference was found between the before and after task phases only when the agent did not provide help. These findings indicate that when the agent explicitly claimed to have helped, empathy levels were maintained at their pre-task levels. In contrast, empathy toward the agent declined when the agent did not make such a claim.

### Willingness to help agents and continuous use of agents

The willingness to help agents results showed a main effect for the agent help factor. As shown in Fig 7(e), participants’ willingness to help the agents increased significantly when the agents had helped them. The results for continuous use of agents also showed a main effect of the agent help factor. As shown in Fig 7(f), participants were more willing to continue using the agent when it had provided assistance. These results are independent of the individual identification of the agent, suggesting that explicitly communicating helpful behavior enhances overall evaluations of the agent.

## Discussion

### Supporting hypotheses

One effective approach to fostering trust and empathy between humans and agents is through the principle of reciprocity. Trust and empathy are essential for the societal acceptance of agents. This study aimed to explore the conditions under which people improve trust and empathy toward agents. Specifically, we focused on whether individuals distinguish between agents based on visual cues (e.g., color) and how the presence or absence of agent-provided help influences trust and empathy. To examine these questions, four hypotheses were formulated and tested using data collected from an experiment. The results did not support H1 and H2, as there was no statistically significant effect of the agent individuality factor. These findings suggest that participants may not have perceived color-based differences as indicative of agent individuality, thereby treating all agents as functionally identical.

In contrast, H3 and H4 were supported by the ANOVA results. For trust, a significant interaction was observed between the agent help factor and the task phase (before vs. after). Simple main effects indicated that participants reported higher levels of trust when the agent explicitly stated that it had provided help. Furthermore, trust in the agent increased when the agent outperformed the participant in the typing task, regardless of whether the agent claimed to have helped. These findings suggest that both explicit prosocial communication and superior task performance can independently enhance perceived trust.

A similar pattern was found for empathy. The simple main effect analysis revealed that empathy levels remained stable after the task when the agent explicitly communicated it had helped. However, when the agent did not convey this, participants’ empathy toward the agent significantly declined. These results indicate that explicitly communicating help is crucial not only for promoting trust but also for maintaining empathy in human-agent interactions.

### Strengths and novelties

One of the strengths of this study lies in the finding that the identification of individual agents—based solely on visual cues such as color—did not produce statistically significant differences in trust or empathy. Although Hypotheses 1 and 2 were not supported, this result offers a notable contrast to reciprocity observed in human–human interactions, suggesting that people may not evaluate agent individuality in the same way as they do human individuality. When agents appear visually similar, even with differences in color, individuals may generalize their trust and empathy from one agent to others that resemble it. This finding suggests that in environments where many visually similar agents coexist, positive interactions with one agent may enhance perceptions of others—regardless of visual distinctions.

Additionally, although not explicitly tested as a hypothesis, the results suggest that merely communicating the agent’s willingness to help enhances participants’ willingness to assist the agent and their intention to continue using it. This finding indicates that even minimal prosocial cues from an agent—such as a simple statement of assistance—may activate cooperative intentions in humans. The increased willingness to help may reflect an altered application of the reciprocity norm when interacting with non-human agents. While the principle of reciprocity has been regarded as a robust social norm across human interactions, the present findings suggest that such reciprocity may not extend naturally to human-agent relationships. This raises important theoretical implications: reciprocal trust and empathy may require more than simple supportive behavior from agents, especially in contexts where their agency and intentionality are not clearly perceived.

Moreover, willingness to continue interacting with the agent may be viewed as a form of empathic behavior toward the agent. This aligns with the concept of empathic agents described in Paiva’s study [39], and is supported by the observed trend in this experiment, where empathy toward the agent increased. While support for Hypothesis 3 was expected, the finding that agent-provided help enhances trust remains meaningful. Importantly, this effect appears to be moderated by the agent’s task performance—in this case, typing ability—which may enhance perceived competence. In contrast, the result supporting Hypothesis 4 is more novel: simply informing the participant that the agent had helped, without elaborate dialogue or emotional display, was sufficient to maintain empathy. Previous research [43, 44] has shown that empathy typically declines in the absence of meaningful conversation or emotional cues, yet this study suggests that even minimal informative feedback about agent support can sustain empathy in human-agent interactions.

### Limitations

A limitation of this study is the reliance on agent appearance—specifically color variation—as a means of individual identification. The findings suggest that participants did not strongly consider color differences when distinguishing between agents. This indicates a need to revisit the design of agent individuality, potentially by incorporating more distinctive visual or behavioral characteristics to enhance perceived differentiation. Additionally, while participants were asked to rate their willingness to help the agent on a 0–100 scale (i.e., the extent to which they would be willing to complete the agent’s typing task), this measure relied on hypothetical responses. It remains possible that participants’ actual behavior—and their trust and empathy toward the agent—would differ if they were required to perform additional typing work in practice. Although the analysis of willingness to help was conducted using ANOVA, the data exhibited a large standard deviation across participants. We considered standardizing the data but ultimately decided against it, as the scale was intended to capture individual variation in willingness to perform additional randomized typing on behalf of the agent.

Furthermore, this study focused only on short-term trust and empathy in a controlled experimental setting. It did not account for the dynamics of long-term human-agent relationships, which are common in real-world applications. Future work should therefore explore trust and empathy over extended interactions and in more ecologically valid environments. Moreover, this study did not assess participants’ prior interaction history with AI or robotic agents, such as the frequency or quality of past encounters. These experiences may influence baseline attitudes and responsiveness to agent behavior. Future research should consider incorporating interaction history as a background variable to better understand how trust and empathy improve over time and through repeated engagement. And then, although this study relied on self-report questionnaires to assess trust and empathy, such subjective measures have inherent limitations. Future research should incorporate more objective or behavioral indicators to comprehensively evaluate human perceptions f agents.

Another limitation is that this study did not include explicit attention-check questions to verify participant engagement. However, the primary task itself—typing 150 randomized letters under time pressure—required sustained attention and provided an indirect measure of effort. In future work, we plan to incorporate explicit attention checks to further ensure data quality. Additionally, participant compensation was relatively modest, which may have affected engagement levels and contributed to the small effect sizes observed. While this factor was not directly assessed, it is an important consideration for future experiments seeking to strengthen participant motivation.

## Conclusion

The key challenge in fostering effective human-agent relationships, especially in light of the growing presence of agents in society, is to enhance trust and empathy between humans and agents. When agents perform tasks similar to those of humans, they often demonstrate superior capabilities. Consequently, when an agent provides assistance to a human, the principle of reciprocity may be triggered, and the person improves trust and empathy toward the agent who has been helped. This study aimed to investigate the factors that influence trust and empathy toward agents. We conducted an experiment using a three-factor mixed design. The between-participant factors were agent individuality (distinguishable vs. indistinguishable) and agent help (provided vs. not provided), while the within-participant factor was task phase (before vs. after). The dependent variables were trust and empathy toward the agent. The results showed an interaction between the agent’s help factor and the before/after task factors. Trust and empathy toward the agent increased when the agent explicitly communicated that it had helped them, compared with when the agent did not help the person. In addition, participants did not perceive the agents as distinct individuals, even when their appearance (e.g., color) differed.

## Supporting information

S1 File Complete data set. (XLSX)
